# The Biological Effects of Novel Nutraceuticals with Curcuminoids and Other Plant-Derived Immunomodulators and Pre-Probiotics

**DOI:** 10.3390/pharmaceutics13050666

**Published:** 2021-05-06

**Authors:** Adina Elena Răducanu, Bianca-Maria Tihăuan, Ioana Cristina Marinaș, Oana Teodora Ciupercă, Carmen Elena Țebrencu, Elena Ionescu, Tatiana Onisei

**Affiliations:** 1 Research and Development Department, The National Institute for Research and Development in Food Bioresources, Str. Dinu Vintilă, No.6, 021102 Bucharest, Romania; adina.raducanu@bioresurse.ro (A.E.R.); tatiana.onisei@bioresurse.ro (T.O.); 2 Research Institute of the University of Bucharest—ICUB, 91–95 Spl. Independentei, 50567 Bucharest, Romania; ioana.cristina.marinas@gmail.com; 3 Physico-Chemistry Laboratory, National Institute of Research & Development for Food Bioresources—IBA Bucharest, 5 Băneasa Ancuța, 020323 Bucharest, Romania; 4 Faculty of Chemical Engineering and Environmental Protection “Cristofor Simionescu”, “Gheorghe Asachi” Technical University of Iași, 73, Prof. Dr. Docent D. Mangeron Street, 700050 Iași, Romania; oana.ciuperca@plantavorel.ro; 5 Research and Processing Centre for Medicinal Plants “PLANTAVOREL” S.A., 46, Cuza Voda Street, 610019 Piatra Neamţ, Romania; carmen@plantavorel.ro (C.E.Ț.); elenaionescu@plantavorel.ro (E.I.); 6 Academy of Romanian Scientists, 54, Splaiul Independentei, 050094 Bucharest, Romania

**Keywords:** novel nutraceuticals, plant-based immunomodulators, pre-probiotics, antimicrobial, cytotoxic

## Abstract

An effective and well-balanced immune system is pivotal for maintaining health. Diet and nutrition can affect the functioning of numerous immune parameters, with direct repercussions on homeostasis. Since our immune functions are indispensable in defending the body against pathogens and thus play a vital role in maintaining health, modulating immune response may well serve as the basis for the development of plant-based functional foods and novel nutraceuticals. This concept is currently utilized in attempts to prevent or mitigate inflammatory reactions via the development of targeted food products or active ingredients since an extended number of phytoconstituents (such as curcuminoids) are associated with beneficial effects on immunity. Immunomodulatory plant-based dietary supplements are considered effective in improving immune functions and reducing the incidence of immunological disorders or imbalances. Therefore, the main focus of this study was to evidence the beneficial biological effects such as antioxidant and antimicrobial, as well as nutritional status, biocompatibility and cell proliferation capacity and immunomodulation of two novel nutraceuticals. The first nutraceutic was based on curcuminoids and other actives from *Trigonella foenum- graecum* (seeds), *Chelidonium majus* L. (aerial parts), *Taraxacum officinale* L. (roots), vitamins (C, D3, A, E) and minerals (zinc) whereas the second one was made of probiotics such as *Lactobacillus acidophilus* and *Bifidobacterium animalis subsp. Lactis* combined with actives from *Helianthus tuberosus* (tubers) and *Psyllium*/*Plantago ovata* (husk) as herbal prebiotics.

## 1. Introduction

Our immune system acts as a protective surveillance grid being able to interpret changes in the world around it and respond appropriately. At its finest, it is capable of distinguishing self from non-self and distinguishing harmless non-self from dangerous non-self. During recent years, the implication of nutrition and dietary supplementation on boosting the immune system capacities has gained interest and might become even more important in the prevention of disease when the interplay between nutritional processes and immune system is better understood. Therefore, nutritional immunology is a rapidly developing field [1,2,3,4].

Studies over the past decade bear out a direct correlation between diet and nutrition on the functioning capacity of numerous immune parameters. This concept is currently utilized in attempts to prevent or mitigate inflammatory reactions via the development of targeted food products or ingredients. Therefore, food and nutrition in general play a critical role in supporting and maintaining immune homeostasis for various groups of individuals [5,6,7,8].

Nevertheless, the research into the possible role of functional foods and nutraceuticals in mitigating immune function is still in its infancy. Therefore, controversies around health claims will remain the rule rather than the exception, especially for nutraceutical compounds [9]. Even though the accurate definition of nutraceuticals is not yet quite well outlined, it can be said that nutraceutical compounds are health-enhancing products that improve mental and physical activities of the body. Their potential role in minimizing risk factors for various diseases are key factors of their success in commercialization [10]. Nutraceutical products are simply a hybrid between drug and food [11].

On the other hand, this terminology is a broader term that includes minerals, vitamins, amino-acids, botanicals or herbs [12], therefore, both dietary supplements and fortified foods can be classified as nutraceuticals [13]. The terminology of nutraceutical was defined by the foundation for innovation in medicine in (New York, USA) in 1989 [11]. Defelice’s definition in 1995 was: “A food or parts of food that provide medical or health benefits, including the prevention and/or treatment of disease” [14]. This term originated from two terminologies: “nutrition” and “pharmaceutical” [11].

As we know by now, modulation of the immune system refers to a certain alteration in the immune response in the form of stimulation, amplification, expression or inactivation of some stage of the immune response. Therefore, an immunomodulator is a material used to induce an effect on the immune system. As a general rule, a product (active ingredient, dietary supplement, etc.) which exhibits a modifying immune system response to a threat, is an immunomodulator. They modulate and arm the immune system by keeping them in a highly prepared state against any threat [15,16].

Modulating immune responses may serve as the basis for the development of functional foods, although constant research on better understanding physiological, immunological and biochemical mechanisms associated with nutrients is necessary [17]. This approach can be a potential route; nevertheless, it is essential to keep in mind that functional foods and nutraceuticals should be regarded as food products with specific health benefits, above those of average consumption levels and aiming at the specific needs of individuals [8,17,18,19].

Over the last few years, a plethora of dietary supplements with natural actives have been widely investigated for their possible use for immunomodulation via various food components, including probiotics, prebiotics, polysaccharides, vitamins, minerals, fatty acids, peptides, amino acids or polyphenols [20]. Moreover, the scientific literature supports the correlation between use of immunomodulatory and probiotic ingredients with prevention of various diseases [1,21,22,23].

Therefore, the main focus of this study was to assess the immunomodulatory properties of two novel nutraceutical formulations, one based on curcuminoids and other actives from *Trigonella foenum- graecum* (seeds), *Chelidonium majus* L. (aerial parts), *Taraxacum officinale* L. (roots), vitamins (C, D3, A, E) and minerals (zinc) and one with probiotics such as *Lactobacillus acidophilus* and *Bifidobacterium animalis subsp. Lactis* combined with actives from *Helianthus tuberosus* (tubers) and *Plantago ovate* (husk) as herbal prebiotics.

## 2. Materials and Methods

### 2.1. Phytochemical Screening of Selected Plant Actives for Novel Nutraceuticals

In order to obtain the novel nutraceuticals, a phytochemical screening of actives was performed on the following species: *Trigonella foenum- graecum* (seeds), *Chelidonium majus* L. (aerial parts), *Taraxacum officinale* L. (roots), *Helianthus tuberosus* (tubers) and *Psyllium/Plantago ovata* (husk).

For the species *Trigonella foenum- graecum* (seeds), *Chelidonium majus* L. (aerial parts), *Taraxacum officinale* L. (roots), first were assessed the humidity (method from Romanian Pharmacopoeia 10th Edition—IX.C.15), granulometry, density and flow, and secondly, qualitative, semiquantitative and quantitative physico-chemical assays were performed in order to highlight the presence of active principles with immunomodulatory potential.

Qualitative determination of saponins, aminoacids, phenolic acids and flavones was performed by HPTLC (high performance thin layer chromatography). For the semiquantitative determination of diosgenin from *Trigonella foenum*—*graecum* (seeds) a HPTLC-UV densitometric method was used. Quantitative assessment of total chelidonine alkaloids from *Chelidonium majus* L.(aerial parts) was performed by UV-VIS spectrophotometry [24].

The species *Helianthus tuberosus* (tubers) and *Psyllium/Plantago ovata* (husk) were analysed in order to identify and quantify the phytocompounds with prebiotic action. Therefore, the qualitative evaluation of saccharides in these two herbal prebiotics was performed using specific tests (the reaction with thymol) and HPTLC identification. The presence with high and medium intensity of saccharides was determined. The HPTLC evaluation of *Helianthus tuberosus* (tubers) evidenced the presence of mono and oligosaccharides such as D-fructose, D-lactose, D-galactose, D-sucrose, D-glucose and inulin. The quantitative evaluation of *Helianthus tuberosus* tubers using UV-VIS spectrophotometric determination revealed a content of total sugars expressed in fructose of 15.62% g/g. These results were relevant for the obtaining of innovative formulas between *Helianthus tuberosus* tubers and *Psyllium* husk [25]. The action of these herbal prebiotics in association with probiotics can ensure the maintaining of a normal intestinal function and health.

### 2.2. Formulas of the Novel Nutraceuticals with Immunomodulatory Properties

The two novel nutraceuticals, one based on curcuminoids and other actives from *Trigonella foenum*—*graecum* (seeds), *Chelidonium majus* L. (aerial parts), *Taraxacum officinale* L. (roots), vitamins (C, D3, A, E) and minerals (zinc) and one with probiotics such as *Lactobacillus acidophilus* and *Bifidobacterium animalis* subsp. *Lactis* combined with actives from *Helianthus tuberosus* (tubers) and Psyllium/*Plantago ovate* (husk) as herbal prebiotics, were obtained in the laboratory of Research and Processing Centre for Medicinal Plants PLANTAVOREL S.A., Piatra-Neamţ, Romania. The formulas of these nutraceuticals resulted from the performed formulation tests. These tests had as a result the selection of an optimal formula capable to ensure a content of phytocompounds which can generate an immune response.

The optimum formula for the tested Immunomodulatory product consisted of 80% actives from which 18% represents curcumin extract with curcuminoids 95% and 20% inactive ingredients, whilst the Pre-Probiotic product formula had 95% actives and 5% inactive ingredients.

To start with a successful formulation for a novel nutraceutical, there are factors to consider such as: an appropriate dosage of the ingredients in order to guarantee the efficiency, validation by scientific studies, and the novelty element of the proposed formula. Thus, the selection of the species *Trigonella foenum- graecum* (seeds), *Chelidonium majus* L. (aerial parts), *Taraxacum officinale* L. (roots) as herbal active ingredients in a immunomodulatory formula is the right choice for obtaining a novel nutraceutical [24,26,27,28,29,30]. In order to provide enough nutrients to meet immune systems requirements and to modulate various physiological functions, curcuminoids, vitamins and minerals can be used. Hence, the combination of *Trigonella foenum- graecum* (seeds), *Chelidonium majus* L. (aerial parts), *Taraxacum officinale* L. (roots) with curcumin extract, C, D_3_, A, E vitamins and minerals such as zinc represents the novel formula for a dietary supplement with immunomodulatory effect. The presence of micronutrients in the novel formula improves the bioavailability of the product. For the proper conditioning of this formula, inactive ingredients (excipients and adjuvants) were used in proportions to ensure an optimal flow of the mixture.

The gut immune system is influenced by many factors, including dietary components and commensal bacteria. Strategies that try to restore the normal gut microbiota have been extensively studied and new therapeutic approaches have appeared and are represented by probiotics (live microorganisms) associated with herbal prebiotics. *Helianthus tuberosus* (tubers) and *Psyllium/Plantago ovata* (husk) are an consistent source of fibers and important herbal prebiotics [25,31,32,33,34] that mixed with probiotics from genus *Lactobacillus acidophylus* and *Bifidobacterium animalis* confer the health of the intestine. This pre-probiotic formula for a plant-based dietary supplement ensures health benefits on the intestinal microenvironment.

### 2.3. Health Claims and Nutritional Status

According to Regulation (EC) No 1924/2006 (nutrition and health claims made on foods), (EU) No 432/2012 (vitamins and minerals), and EFSA regulations regarding health claims of botanicals, there are several health claims associated with ingredients used in our two nutraceuticals. Their importance is underlined by legislation support, as it facilitates rapid association of active and more efficient development of formulas.

The health claims [35,36] of our main ingredients support our formula’s rationale, and for the Immunomodulatory nutraceutical, they are:

*Chelidonium majus*: contributes to the maintenance of normal liver function and additionally supports the digestion and the body’s purification—ID 2238.

*Curcuma longa*: curcumin and its derivatives are currently used as oral supplements in the management of various dermatological and medical conditions [37]; moreover, it helps maintain the efficacy of the immune system and to maintain resistance to allergies. It also has significant antioxidant properties—ID 4009; According to ID 2751, curcuma prevents the accumulation of fats and facilitates their clearance by the liver, maintaining the health of the liver—ID 3098; is registered to provide cell protection—ID 3748; also helps maintain the health of joints and bones—ID 4012, keeps the skin healthy—ID 4008 and supports heart function and blood circulation—ID 4010;

*Trigonella foenum-graecum*: main benefits are in providing support and balance to the metabolism of fats, contributing to normal glucose and insulin metabolism—ID 4186; it helps with the physiological pH balance of the stomach—ID 3623, through balanced diets helps the control of lipidic metabolism (cholesterol and triglycerides)—ID 3832; Antioxidants can protect from free radicals and helps in case of foods intake deficiency or increased amount of nutrients. Protection against the free radicals action due to stress, alcoholics, UV exposure or polluted ambiance conditions—ID 3935; is also is a physical and mental tonic—ID 4494.

*Taraxacum officinale*: confers protection against free radicals action due to stress, alcoholics, UV exposure or polluted ambiance conditions—ID 3828, helps maintain urinary tract function by maintaining normal urinary flow—ID 3827; It has prebiotic activities, contributes to the gastrointestinal well-being by its prebiotic effects—ID 4315; also helps the physiological pH balance of the stomach—ID 3609.

According to (EU) No 432/2012, vitamins C, E, A, D and zinc all contribute to the normal function of the immune system and to the protection of cells from oxidative stress [38].

As for the Pre-Probiotic formula:

Topinambur/*Helianthus tuberosus*: is actively implicated into the growth, development and maintenance of body function—ID 4412; A generally restorative product for strengthening of the immune system, activates body’s antioxidant protection system, increases body’s resistance against the harmful effects of the environment, infections and other unfavorable factors—ID 2449.

Psyllium/*Plantago ovata*: its main effect are correlated to lowering cholesterol and blood lipids level—ID 4461; It helps to control blood levels of cholesterol—ID 2106, contributes to intestinal transit and intestinal function and helps to maintain a healthy bowel and facilitate intestinal transit—ID 3932.

### 2.4. Evaluation of Key Physico-Chemical Parameters and Antioxidative Status

(a) Sample preparation

The nutraceuticals were solubilized in 70% ethanol to obtain a final concentration of 10 mg dry product/mL. The extracts were obtained by an ultrasound-assisted method for 20 min. The method involves the use of ultrasounds, with frequencies ranging from 20 kHz to 2000 kHz, allowing cell lysis. The extract thus obtained was centrifuged for 15 min at 10,000 rpm to remove insoluble material.

(b) Total phenol content

The total phenol content was measured spectrophotometrically by the method described by Singleton and Rossi in 1965 and modified by other authors [39,40]. Over 0.1 mL of sample or standard solution (gallic acid) was added 0.9 mL of H_2_O d.d. After adding 0.1 mL of Folin Ciocâlteu reagent, stir for 5 min, then add 1 mL of 7% Na2CO3 and make up to a final volume of 2.5 mL with H_2_O d.d. After incubation at room temperature in dark place for 60 min, the absorbance values at 765 nm were recorded. The calibration curve was performed for gallic acid concentrations between 0–300 μg/mL (R^2^ = 0.9969).

(c) Total flavonoid content

The total flavonoid content was determined by the aluminum chloride method [41] which consisted of treating 0.1 mL of sample/standard solution (quercetin) with 0.1 mL of 10% sodium acetate. A total of 0.12 mL of 2.5% AlCl_3_ solution was added to this mixture and made up to a final volume of 1 mL with 70% ethanol. After stirring, this solution was allowed to stand for 45 min in the dark and the absorbance of the mixture was read at 430 nm. The calibration curve was performed for quercetin concentrations in the range of 0–200 μg/mL (R^2^ = 0.996).

(d) DPPH

The measurement of DPPH radical uptake activity was performed by an adapted method after Madhu, 2013 [42]. The reaction mixture consisted of adding 100 μL of sample/standard and 100 μL of 0.3 mM DPPH radical solution in 50% ethanol. The absorbance reading was performed at λ = 517 nm after 30 min of reaction, using a UV-VIS spectrophotometer. Ethanol was used as a control. The concentrations used for the Trolox calibration curve were in the range of 5–80µM (R^2^ = 0.9968).

(e) CUPRAC

The CUPRAC method is based on the reduction in the cupric complex, neocuproine (Cu (II) -Nc) by antioxidants in cuprous form (Cu (I) -Nc). Copper ion reduction was performed according to a method adapted from Meng et al., 2011 [43], as follows: 20 μL of sample/standard solutions were mixed with 60 μL CuSO4 (5 mM), neocuproine 60 μL (3.75 mM) and distilled water (560 μL), reaching a final volume of 700 μL. After 30 min, the absorbance was measured at 450 nm. The standard Trolox solutions required for the calibration curve were between: 1–0 mM (R^2^ = 0.9962).

(f) FRAP

The FRAP method is based on the ability of antioxidants to reduce the tripyridyltriazine-Fe^3+^ (Fe (III) -TPTZ) complex to the blue-colored tripyridyltriazine-Fe^2+^ (Fe (II) -TPTZ) complex. The determination of the antioxidant power of iron reduction was performed by the method described by Benzie and Strain, 1999 [44] with some modifications [45]. The following solutions were prepared: 300 mM acetate buffer, pH 3.6, 10 mM TPTZ stock solution in 40 mM HCl and 20 mM FeCl_3_ solution in distilled water. Prepare the FRAP reagent by mixing 300 mM acetic acid-sodium acetate buffer, pH 3.6 with 10 mM TPTZ solution and 20 mM FeCl_3_ solution (10:1:1). The FRAP reagent is kept on a water bath at 37 °C until the time of analysis. At 10 µL sample/standard solution, 190 µL FRAP reagent and incubate for 30 min at 37 °C. After incubation, the absorbance at λ = 593 nm is read. The calibration curve was performed for the concentration range 50–250 µM Trolox/mL (R^2^ = 0.9938).

### 2.5. Assessment of Antimicrobial Activity

Evaluation of antimicrobial activity of samples was performed by determining the logarithmic and percentage reduction in reference microorganisms.

Three reference strains from the American Type Culture Collection (ATCC, Manassas, VA, USA) were used for testing (*Staphylococcus aureus* ATCC 6538, *Escherichia coli* ATCC 8739, and *Candida albicans* ATCC 10231). The microbial suspensions of 1.5 × 10^8^ CFU/mL, obtained from fresh 15 to 18 h cultures, developed on solid medium, were adjusted according to McFarland standard 0.5, respectively, McFarland 1 for microfungi, and then serially diluted to 10^5^. Samples were weighed and the inoculum volume was adjusted according to the mass of the samples. The samples were placed in contact with the microbial inoculum for 30 min, thoroughly spun on a vortex, and afterwards 5 decimal serial dilutions were carried out in order to determine the logarithmic and percentage reduction in the microbial populations. A total of 10 µL in triplicate were inoculated in spot on Muller Hinton solid medium, respectively, Sabouraud for microfungi. After 18–24 h of incubation at 36 ± 2 °C the plates were read by counting the colonies.

The logarithmic reduction was calculated using the formula:

Logarithmic reduction = lg A/B

A = no. of viable organisms before treatment; B = no. of viable organisms after treatment

The percentage reduction in microbial populations was calculated using the formula:

P = (1–10 ^−L^) × 100

where P is the percentage reduction and L the logarithmic reduction

### 2.6. Determination of Intracellular Toxicity

The L929 murine fibroblasts (ECACC—European Collection of Authenticated Cell Cultures) were selected as a model for intracellular toxicity assessment of novel products. L929 cells were cultivated in DMEM (Dulbecco’s Modified Eagle Medium, Sigma-Aldrich) media supplemented with 10% FBS (Fetal Bovine Serum, Sigma–Aldrich) and 1% Pen/Strep (penicillin/streptomycin solution, 50 µg/mL, Sigma–Aldrich) for 24 h at 37 °C, 95% humidity with 5% CO_2_. After 24 h, the cells were washed with PBS (Phosphate Buffered Solution, Sigma–Aldrich), harvested using trypsin (Sigma–Aldrich) and counted using Trypan Blue (Sigma–Aldrich, St. Louis, MO, USA) and a hemocytometer. The seeding density for the MTT and LDH assays was optimized at 5 × 10^4^.

Samples were prepared by a conventional heating technique in PBS. This mixture was mechanically stirred until it was well homogenized, then heated to extraction temperature (i.e., 35–40 °C) and rigorously stirred throughout the extraction time (i.e., 5–150 min). The extracts were then carefully filtered using a vacuum pump filter.

The MTT assay evaluates the cellular metabolic activity, therefore is a good indicator of cell viability, proliferation and cytotoxicity. This colorimetric assay is based on the reduction in a yellow tetrazolium salt (3-(4,5-dimethylthiazol-2-yl)-2,5-diphenyltetrazolium bromide or MTT) to purple formazan crystals by metabolically active cells [46,47].

Cells seeded at 5 × 10^4^ density in a clear 96 well cell culture plate were treated with the novel products in the following concentrations: 200 mg/mL, 100 mg/mL 25 mg/mL, 3.12 mg/mL, 0.2 mg/mL and 0.01 mg/mL. Afterwards, cells were incubated for 24 h at 37 °C, 95% humidity with 5% CO_2_. After 24 h of exposure to novel products, cells were incubated for 4 h with MTT reagent (Roche) at 37 °C, 95% humidity with 5% CO_2_. After incubation, cells were treated with MTT solvent (Roche) for 15 min at room temperature. Absorbance was measured using FlexStation 3 (Molecular Devices Company, Sunnyvale, CA, USA) at OD = 570 nm.

The LDH assay involves the assessment of cell death by quantification of plasma membrane damage. This increase in the amount of enzyme activity in the supernatant directly correlates to the amount of formazan formed. Therefore, the amount of color formed in assay is proportional to the number of lysed cells [47]. With the LDH Cytotoxicity Detection Kit (Roche, Basel, Switzerland), LDH activity was measured in culture supernatants using FlexStation 3 (Molecular Devices Company, Sunnyvale, CA, USA) at 492 nm with a 600 nm wavelength reference. Cells were prepared and treated alike as for the MTT assay.

### 2.7. Evaluation of Immunomodulatory Activity

To investigate the immune-modulatory activity of the novel compounds, RAW murine macrophages were cultivated in Dulbecco’s Modified Eagle’s medium (DMEM) supplemented with 10% FBS. Compounds were diluted 1:5 in the cell culture medium and the stimulation of macrophages was done for 24 h. Supernatants were collected and used for cytokine quantification. Murine IL-10 and IL-6 were quantified using ELISA (Thermo Scientific, Waltham, MA, USA) following the manufacturer’s instructions.

### 2.8. Statistical Assesment of Data

All tests were performed in triplicate and the results were expressed as ±SD (standard deviation). The statistical analysis was performed by Unpaired *t*-test (*n* = 2) using the GraphPadPrism Software (San Diego, CA, USA).

## 3. Results

### 3.1. Evaluation of Key Physico-Chemical Parameters and Antioxidative Status

The flavonoid content represents approximately 14.21% of the total polyphenols for the immunomodulatory product and 2.85% for the pre- and probiotic product (Table 1). In fact, the polyphenol content determined by the Folin Ciocalteu method probably comes from the following ingredients: Chelidonium majus (rich in quercetin, caffeic acid and neochlorogenic acid) [48], Trigonella foenum-graecum (apigenin, luteolin and kaempferol as aglycones and hydroxycinnamic acids mostly dominated by caffeic acid derivatives) [49], Curcuma longa (chlorogenic acid, curcuminoids, isorhamnetin, digalloyl-hexoside) [50] extract and vitamin C for the first product while for the second product the continuum of polyphenols and flavonoids, respectively, is given by Helianthus tuberosus (chlorogenic and dicaffeoylquinic acids) [51] and Plantago ovata (gallic acid and rutin) [52].

Flavonoids are secondary metabolites of plants, with numerous pharmacological functions: antioxidant, antimutagenic, antibacterial, anti-inflammatory, antiallergenic, enzymatic modulators and antitumor. These phytocompounds are in the form of free aglycones or in the form of glycosides. Flavonoids are classified into flavones, flavanols, isoflavones, flavonols, flavanones, flavanonols and chalcones. The various structures of flavonoids have led to numerous modulating properties on the immune system response. These compounds can act both synergistically and antagonistically [53].

The Pre-/Probiotic product has a mixture of plant materials poor in phenolic compounds, so it correlates with reduced antioxidant capacity.

The higher values obtained by the DPPH method highlight the double mechanism involving both proton transfer (HAT) and electron transfer (SET) in the case of the immunomodulatory product [54]. Meanwhile, the pre- and probiotic product involves a predominantly SET antioxidant mechanism, but considerably lower than the immunomodulatory one (Table 2).

The evaluation of the antioxidant activity of phenolic compounds is performed mainly by chemical methods. Antioxidant testing methods, including Iron Reducing Antioxidant Power (FRAP), Copper Reducing Antioxidant Capacity (CUPRAC), 2,2-diphenyl-1-picrylhydrazyl (DPPH) and photochemiluminescence have been developed as rapid assessment and screening tools. According to the literature data [48,55,56,57], it has been observed that the antioxidant activity of the immunomodulatory product is higher than the extracts from individual plant materials, thus, the components act synergistically. None of these in vitro methods are relevant to the physiology itself in vivo. Phenolic compounds generally have low bioavailability. Cellular determinations can provide physiologically relevant information. However, the rapid metabolism and low solubility of phenolic compounds often lead to low bioavailability in vivo. The plasma concentration of phenolic compounds is generally low, generating insufficient antioxidant activities for a significant effect [58].

### 3.2. Antimicrobial Activity

The methodology for evaluating antimicrobial activity aims to assess the safety and effectiveness of products. Testing of antimicrobial properties involves testing the ability of microorganisms to survive under the effect of a given antimicrobial agent (or with antimicrobial potential), at a certain concentration and for a certain period of time.

Although our novel products do not aim to obtain an antimicrobial effect after administration, the existence of this quality has a number of advantages, both for the consumer and for the quality of the product.

**Figure 1:**
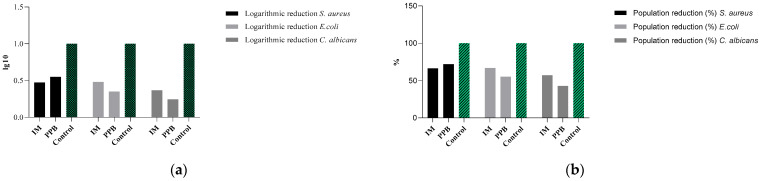
(**a**) Logarithmic reduction and (**b**) Population reduction in *Staphylococcus aureus* ATCC 6538, *Escherichia coli* ATCC 8739, and *Candida albicans* ATCC 10,231 strains by tested samples represented by novel nutraceuticals.

Samples were evaluated in the presence of *Staphylococcus aureus* ATCC 6538, *Escherichia coli* ATCC 8739, and *Candida albicans* ATCC 10,231 strains. The results on logarithmic reduction are shown in Figure 1a. Logarithmic factor 1, which represents a 99% efficiency in bacterial reduction, was considered as a control. For the Immunomodulator product, in the presence of *S.aureus* ATCC 6538 a reduction of 0.47 log was obtained, and for the Pre-Probiotic product a reduction of 0.55 log, this having a more pronounced antimicrobial effect.

In the case of Gram-negative strain *E. coli* ATCC 8739, results show a logarithmic reduction of 0.48 log for the Immunomodulatory product and of 0.35 log for the Pre-Probiotic product. Both products also significantly reduced *E. coli* populations (55–66%) (Figure 1b).

The reduced efficiency of the two products on the Gram-negative *E. coli* strain may be due to the structural differences between Gram-positive (are more susceptible to antimicrobial treatments) and Gram-negative microorganisms (presence of outer membrane, lipopolysaccharides, murein, etc.).

The logarithmic reduction (Figure 1a) and the percentage reduction (Figure 1b) in the case of the fungal strain *C. albicans* ATCC 10,321 is modest, the Immunomodulatory product being more efficient (0.36 log and 57.1% reduction).

### 3.3. Determination of Intracellular Toxicity

The analysis of the biological action on the in vitro cell model of the two novel products was performed by evaluating cellular viability, proliferation and membrane integrity by MTT and LDH assays.

**Figure 2:**
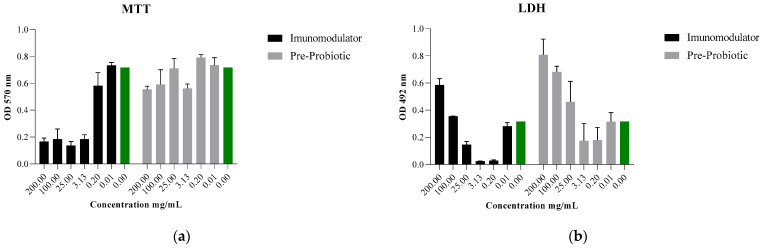
(**a**) MTT and (**b**) LDH assessment of novel nutraceuticals on L929 murine fibroblasts.

In the case of the Immunomodulatory product, concentrations of 200 mg/mL, 100 mg/mL and 25 mg/mL significantly reduced the percentages of cell viability (Figure 2a,b). Lower concentrations favor cell viability and proliferation. In the case of the Pre-Probiotic product, the percentages of cell viability are high, all the concentrations tested do not negatively influence the viability and cell proliferation.

The MTT test (Figure 2a) performed on the L-929 fibroblasts highlights, in the case of the Immunomodulator product, the cytotoxicity of the concentrations 200 mg/mL, 100 mg/mL and 25 mg/mL; the decrease in the concentrations of this product is directly proportional to the increased viability rate. The Pre-Probiotic product favors cell proliferation, having at lower concentrations the effect of a nutritious substrate, the viability of the cells not being affected.

Evaluation of cytotoxicity by LDH test (Figure 2b) confirms the results obtained by MTT test; in the case of the Immunomodulatory product, the administration of concentrations higher than 25 mg/mL increases the degree of toxicity at the cellular level.

### 3.4. Evaluation of Immunomodulatory Activity

The immune system is a remarkably developed defense system which guards against attacking factors. It is capable of producing diverse molecules and cells able to distinguish and reduce unlimited changes from external and unwanted agents [17].

IL-10, an important anti-inflammatory cytokine, plays a critical role in the control of immune responses, it opposes the switch to the metabolic program induced by inflammatory stimuli in macrophages [59]. It is notable that IL-10 effectively down-regulates proinflammatory cytokines, such as IL-1, IL-6, and TNF-α [60]. As for IL-6 (pro- and anti-inflammatory cytokine), is a very interesting, pleiotropic cytokine with complex roles in inflammation and metabolic disease [61]. Stenvinkel, P et.al., mention that bio-incompatibility and oxidative stress are factors that may be associated with increased circulating IL-6.

**Figure 3:**
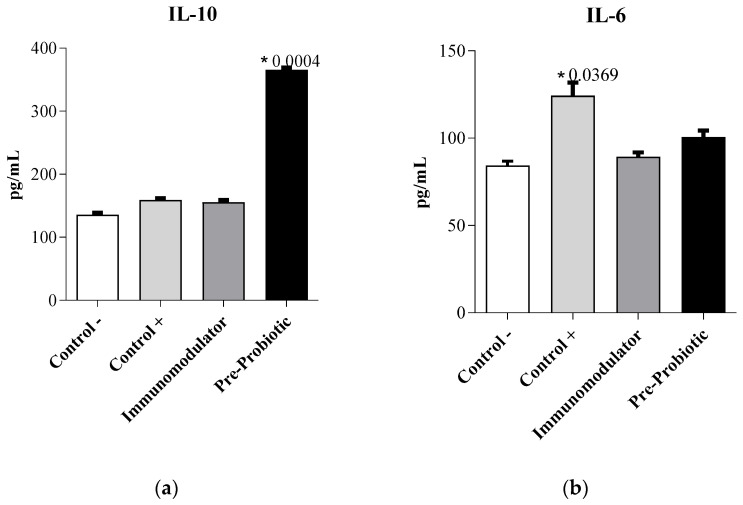
Levels of anti-inflammatory (**a**) IL-10 cytokine and pro-inflammatory (**b**) IL-6 cytokine of RAW murine macrophages treated with novel formulated products. Control − are untreated cells and Control *+* is positive control of cells treated with a suspension of *E. coli* 10^6^. * *p* < 0.05.

Our study showed that, after treatment with novel formulated products, the IL-10 levels were significantly increased (Figure 3a), especially by the *Pre-Probiotic* supplement. Conversely, the Immunomodulatory supplement yielded IL-10 levels similar to the control group. IL-6 levels were slightly increased (Figure 3b), compared with the negative control, especially for the *Pre-Probiotic* product.

As mentioned by other authors, coupling aggressive inflammatory factors with the delayed secretion of IL-10 ensures that any inflammatory response will securely be down-regulated after some time. The increased levels of IL-10 and slightly reduced levels of IL-6 showcase good indicators for initial phase immunostimulation.

**Figure 4:**
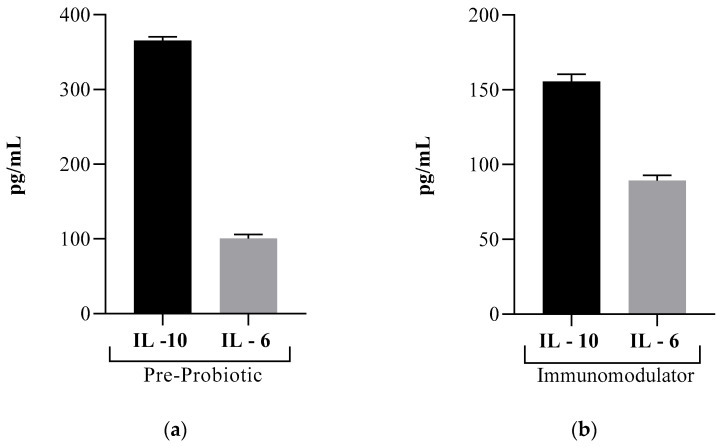
The ratio of IL-6 to IL-10 for the novel formulated products; (**a**) Pre-Probiotic; (**b**) Immunomodulator.

Assessing the ratio of IL-6 to IL-10 can be constructive in evaluating cellular response after exposure to stress or trauma-causing factors [62]. For the Pre-Probiotic product (Figure 4a), we can remark the increased levels of IL-10 and reduced levels of IL-6, indicating good immunostimulation; the potent immunosuppressive cytokine, IL-10 was activated properly and inhibits the production of the proinflammatory cytokine IL-6. As for the Immunomodulator product, keeping in mind that the yielded IL-10 levels were similar to the control group, and the IL-6 levels were slightly higher than the negative control, we can attest a case of delayed secretion of IL-10 and a small-scale immunostimulation.

## 4. Discussion

Immunomodulation via nutrition has become an interesting approach in the research world. Such products modulate and arm the immune system by keeping them in a highly prepared state against any threat.

As stated above and mentioned by other authors as well, there is a controversy over a specific definition and set of regulations to define the nutraceutical compounds, however, nutraceutical compounds are health-enhancing products that improve mental and physical activities of the body [10,14].

This study evaluated two novel nutraceuticals with Immunomodulatory and Pre-Probiotic properties derived from curcuminoids, vitamins, minerals and plant actives from *Trigonella foenum- graecum*(seeds), *Chelidonium majus* L. (aerial parts), *Taraxacum officinale* L. (roots), *Helianthus tuberosus* (tubers) and Psyllium/*Plantago ovata*(husk).

The Immunomodulatory product has a higher content of polyphenols and flavonoids than the Pre- and probiotic one, correlating with the specific ingredients of each product. Thus, the product with a higher content of vegetable ingredients proved to be rich in polyphenols, flavonoids and better antioxidant activity.

Antioxidant activity has been demonstrated by three chemical methods involving different mechanisms of action, but in vitro determinations on eukaryotic cells are needed to highlight reduced intracellular oxygen reactive species concentrations, improved metabolism and cell proliferation.

According to the results obtained from the microbiological analysis, it can be seen that the antimicrobial activity of Immunomodulatory and Pre-Probiotic products is more pronounced against Gram-positive strain *S. aureus* ATCC 6538 and more modest (bacteriostatic/fungistatic) against Gram-negative strain *E. coli* ATCC 8739 and the fungal strain *C. albicans* ATCC 10321. The samples showed moderate efficiency (bacteriostatic/fungistatic) from an antimicrobial point of view. However, further investigations are needed using alternative methods to confirm the effects obtained.

Evaluation of the biological activity on the in vitro cellular model of the Immunomodulatory and Pre-Probiotic products, with the help of MTT and LDH tests, showed percentages of increased viability, the composition and formulation of the products proved to be well balanced and suitable for consumption at lower concentrations. As the results indicate, it should be borne in mind that in the case of the Immunomodulatory product, the cytotoxicity of concentrations of 200 mg/mL, 100 mg/mL and 25 mg/mL is quite high (70% reduction in cell viability); the decrease in the concentrations of this product is directly correlated to the viability rate. The Pre-Probiotic product favors cell proliferation, having at lower concentrations the effect of a nutritive substrate, the viability of the cells not being affected.

As for the immunomodulatory assessment, we know that cytokines are small secreted proteins released by different types of cells with specific effects on cellular signaling and communication via binding to their receptors on the cell surface [15].

The capacity of the Pre-Probiotic product to upregulate IL-10 was demonstrated. As other authors report, pre-probiotics have a beneficial impact on immunomodulation [63], emphasizing the fact that IL-10 (among other cytokines) inhibit pro-inflammatory cytokines, chemokines, and chemokine receptors, responsible for intestinal inflammation [63]. As for the expression of IL-6, studies have revealed that it favors the clonal expansion of IgA B lymphocytes and stimulates the production of antibodies such as IgM, IgG, and reduced secretion of IgE [64].

Recently, Karamese et al. [65] administrated a mixture of *Lactobacillus* and *Bifidobacterium* species to rats for evaluation of the immunomodulatory effects of probiotics. Their study demonstrated that modulation or regulation of immune responses is evident through the upregulation of IL-10 (an anti-inflammatory cytokine) and the downregulation of TNF-α and IL-6 (proinflammatory cytokines). Moreover, it has been reported that the application of probiotics leads to significant increases in IgA and IgG concentrations in rats.

Evaluating the ratio of IL-6 to IL-10 for both products, we observed that the effect of the Pre-Probiotic product was noticed rapidly (at 24 h), whereas it is possible that the Immunomodulator product requires a longer contact time for higher stimulation.

## 5. Conclusions

In conclusion, the novel formulated nutraceuticals present great potential for their designated purposes, also being good candidates for further in vivo studies. The evaluation of effectiveness has proven to be in correlation with some of their associated health claims approved by Regulation (EC) No 1924/2006 on nutrition and health claims made on foods, (EU) No 432/2012 on vitamins and minerals, and EFSA regulations regarding health claims of botanicals. For the *Probiotic* formula, as a cautionary mention, it should be kept in mind that the European Commission has indicated that the term *probiotic* implies a health claim and therefore cannot be used in conjunction with an approved health claim. According to the Guide for the implementation of Regulation 1924/2006 (Chapter III. 1) it is specified that if the name of a substance implies an indication of a functionality or an effect on health, it is considered a health mention. EFSA has so far not approved any health claims for pre and probiotics, so product labels cannot include messages regarding the health benefits of pre/probiotics.

As for future perspectives, further in vivo studies are envisioned. We seek to demonstrate and confirm on suitable animal models (mice) the beneficial effects of obtained products in order to safely place them onto current market. Certainly, preliminary human clinical studies, conducted on a specific group of patients (i.e., patients with Inflammatory Bowel Diseases, or with immuno-sensitive systems (i.e., allergies)) would increase the consumer compliance, sustain associated health claims and diminish the gap between pharma grade products and functional foods. Moreover, all this could lead to assembling dossiers for Novel Food application according to EFSA.

In our vision, as a regulatory organism for dietary supplements placement on the Romanian market, manufacturers need to push forward and contribute more to the research community with additional testing of products. This could benefit both parties, helping improve current legislation and mitigate for higher placement on the therapeutic market of dietary supplements.

## Figures and Tables

**Table 1:** Total content of polyphenols and flavonoids expressed as mg GAE/g sample and mg QE/g sample, respectively.

Sample	Total Polyphenols (mg GAE/g)	Total Flavonoids (mg QE/g)	% Flavonoids from Total Polyphenols
Immunomodulator	144.07 ± 4.21	20.472 ± 2.005	14.21
Pre-Probiotic	1.13 ± 0.26	0.032 ± 0.009	2.85

**Table 2:** Antioxidant activity of the Immunomodulatory and Pre-Probiotic products.

Sample	DPPH (μM Trolox/mg)	CUPRAC (μM Trolox/mg)	FRAP (μM Trolox/mg)
Immunomodulator	2956.63 ± 292.12	1001.13 ± 30.82	918.91 ± 2.97
Pre-Probiotic	7.47 ± 0.04	33.59 ± 0.76	18.25 ± 0.55

## Data Availability

Not applicable.
